# Treatment of Metabolic Syndrome in Children

**DOI:** 10.3389/fendo.2019.00702

**Published:** 2019-10-15

**Authors:** Elena Fornari, Claudio Maffeis

**Affiliations:** Pediatric Diabetes and Metabolic Disorders Unit, Department of Surgical Sciences, Dentistry, and Pediatrics, and Gynecology, University of Verona, Verona, Italy

**Keywords:** obesity, treatment, children, adolescents, metabolic syndrome

## Abstract

The Metabolic Syndrome may be tentatively defined as the clustering of several metabolic risk factors in the same individual. A progressively higher number of children and adolescents is affected by this syndrome worldwide, mainly as a consequence of the constant increase of the prevalence of obesity and sedentary habits. As obesity, the chance that the metabolic syndrome traks into adulthood is high. Moreover, the evidence of an association between the duration of the exposition to metabolic risk factors and morbidity and mortality justifies early treatment and prevention of the metabolic syndrome in both children and adolescents. Treatment includes behavioral interventions, adequate nutrition and physical activity, and, if necessary, pharmacological treatments aimed at reducing excessive weight, dyslipidemia, hypertension, and glucose impairments. A multidisciplinary and staged approach to treatment, which includes pediatrician, mental health practitioner, dietician, and nurses, is crucial. Usually, the reduction of fat mass promotes an overall improvement of all the components of the metabolic syndrome. Nevertheless, every single component of the metabolic syndrome should be treated as quickly as possible, by using the best current practice. Drugs may be necessary for treating hypertension, type 2 diabetes mellitus and dyslipidemia. In selected cases of gross obesity resistant to treatment, surgical therapy may be also performed.

## Introduction

**Figure 1:**
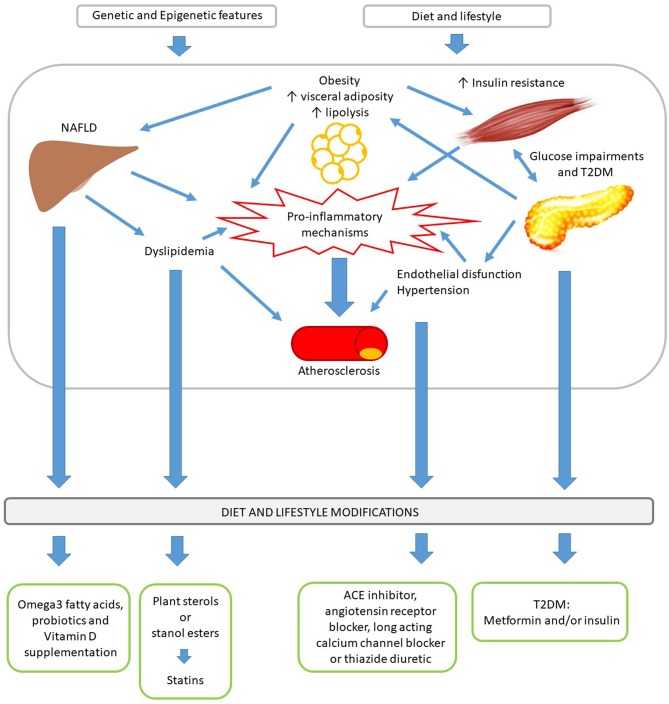
Main MetS pathomechanisms and corresponding first-line approach and treatment.

Regardless of the many different definitions of the Metabolic Syndrome (MetS) in child and adolescent (1–4), early screening and treating the single components, which contribute to its development, have a pivotal role in reducing cardiometabolic risk (5). There is no uniform agreement in how to treat the individual risk factors other than excessive weight management. However, the set of MetS component risk factors share their pathophysiologic origins and many common treatment approaches grounded in lifestyle modifications (Figure 1) (5). The Bogalusa Heart Study demonstrated that every single risk factor increases the development and severity of atherosclerotic lesions (6). The American Academy of Pediatrics (AAP) underlines the importance to treat each risk factor individually, independently from the definition of MetS, with the ultimate goal of reducing cardiometabolic risk (5). In fact, the concept of CVR has to be considered a continuum and not the sum of the overcoming of cut-off points for the single risk factors (5).

Treatment of MetS includes behavioral interventions and, if necessary, pharmacological treatments aimed at reducing excessive weight, dyslipidemia, hypertension, and glucose impairments (Table 1) (7, 8). Once every single risk factor is identified, it should be treated by using the best current practice as quickly as possible. The Princeton Prevalence and Follow-up Studies demonstrated that pediatric MetS predicted adults MetS with an OR of 9.4 (9). Considering that the maintenance of the pathologic conditions until adulthood increases the risk, early treatment is essential (5).

**Table 1:** List of MetS components and corresponding first-line approach and treatment.

**Mets components**	**First-line approach**	**Treatment**
Obesity	Lifestyle interventions: Diet (caloric restriction, specific targets suggested by dietitians) PA (60 min of moderate/vigorous PA every day, including vigorous activity 3 day per week)	PHARMACOLOGIC TREATMENT Orlistat, when indicated SURGICALTREATMENT Bariatric surgery, when indicated
Hypertension	Lifestyle interventions: Diet (reducing sodium, increasing olive oil polyphenols, increasing intake of fruits, and vegetables) PA(30–60 min of moderate/vigorous PA at least 3–5 days per week)	PHARMACOLOGIC TREATMENT Start with a single medication at the low end of dosing range. Titrate every 2–4 weeks. ACE inhibitor, angiotensin receptor blocker, long acting calcium channel blocker or thiazide dieuretic are the first choices
Dyslipidemia	Lifestyle interventions: Diet (reducing total fat between 25 and 30% of daily calories and cholesterol intake <300 mg/day, reducing simple carbohydrate intake, possible use of plant sterols or stanol esters) PA	PHARMACOLOGIC TREATMENT Statins when indicated
Glucose impairments and T2DM	Lifestyle interventions: Diet PA	PHARMACOLOGIC TREATMENT Glucose impairments: the use of metformin is uncommon T2DM: metformin and/or insulin
NAFLD	Lifestyle interventions and weight loss. Probiotics and omega3 fatty acids may ameliorate disease progression. Vitamin E can improve hepatocellular balloning	

*PA, physical activity; BP, blood pressure; T2DM, type 2 diabetes mellitus; NAFLD, non-alcoholic fatty liver disease*.

There is common agreement that obesity prevention and treatment in children and adolescents is “the first-line approach” to reduce the cardiovascular risk (CVR) (8, 10). In fact, prevalence of metabolic syndrome in childhood is increasing in parallel with the increasing trends in obesity rates (11, 12). In addition, although the pathogenesis of MetS is not completely understood, good evidence suggests that interactions between obesity, insulin resistance and pro-inflammatory state, certainly play a key role in its development and maintenance (13). Treatment of obesity and other MetS components share many common elements, so interventions that improve one condition could consequently be useful to ameliorate also the others. For example, weight reduction following non-surgical interventions, including dietary changes, increased physical activity, and behavioral therapy, was found to be associated with improvements in several metabolic parameters such as dyslipidemia and hypertension (14–16). Weight reduction also results in decrease of insulin resistance and low-grade inflammation (17). Combining dietary interventions and physical activity is more effective than either single intervention in reducing BMI (5). In addition, data from a meta-analysis demonstrate that the positive effect of ameliorating unhealthy diets and reducing sedentary behavior in long-term trials was more pronounced when directed toward children compared to adolescents (18). Therefore, early management and treatment are crucial, in particular in pediatric population.

## Dietary Intervention

The mechanisms that explain the correlation between diet modification and effects on MetS components are not fully clear. Lowering intake of simple sugars may reduce pancreatic stimulus for insulin production (5). Caloric restriction reduce mitochondrial substrate availability (19) whereas increased dietary fiber intake decreases the glycemic load (20). The primary goal in dietary intervention programs should focus on reducing total energy intake (21). It is essential that a dietician with proved experience in growing children's needs supervise the caloric reduction (20, 22). Specific targets for dietary habits have also demonstrated efficacy in reducing BMI, i.e., substitution of sugar-sweetened beverages with water (23), portion control education, increased intake of fruits and vegetables, reduction of dietary fats, sodium, and processed foods, increased intake of fiber (8, 20, 24). The AAP Guidelines report the appropriate recommendations about diet and nutrition in the prevention and first-line therapy approach of obesity and other risk factors (8). Specific nutritional interventions for the therapy of each single MetS component will be treated in the respective paragraph of the text. The intervention of an expert multidisciplinary team, which includes dietician, psychologist, and providers with proven experience with pediatric obesity, is much more effective to obtain a modification of eating habits than other approaches (20, 25).

## Physical Activity

Physical activity is any body movement produced by contraction of skeletal muscle, which results in an increase in energy expenditure above the basal level (8). Strong evidences prove the beneficial metabolic effects of reducing sedentary behavior, i.e., increasing walking and physical activity on the overall health of children and adolescents (8, 26–28). In addition, physical inactivity has been identified as an independent risk factor for coronary heart disease in adults (29). Time spent in moderate to vigorous physical exercise is inversely associated with continuous risk score of MetS (30). Therefore, an increase in physical activity, also independently form a change in weight status, represents an important treatment strategy for childhood MetS and results in improving of metabolic parameters and subclinical measures of atherosclerosis (8, 20).

Physical activity increases hepatic mitochondrial substrate metabolism, reducing the availability of substrate for lipogenesis and mitigating insulin resistance (31, 32) and causes mitochondrial biogenesis in liver and muscles (32). There is strong evidence that physical exercise promotes a reduction of fasting insulin levels and decreased insulin resistance in children and adolescents (31). It is helpful in improving lipid profile by increasing HDL concentration and decreasing both LDL and triglycerides concentrations (33). In addition, exercise can result in improvement of endothelial function with reduction in both systolic and diastolic blood pressure, independently from the type of training (aerobic, resistance, or combined) (34). It ameliorates body composition and has an abdominal fat reduction effect (34–36). As activity levels increase, pro-inflammatory cytokines and markers of oxidative stress decrease (31), producing anti-inflammatory effects (37).

Structured physical activity interventions favor decreased daily energy intake in obese adolescents (38). The AAP recommends at least 60 min of moderate-to-vigorous activity every day for children older than 5 years, including vigorous activity on 3 days per week (8). A more conservative approach for both prevention and treatment of obesity and related risk factors has been suggested by the Endocrine Society. They recommend the reduction of inactivity and a minimum of 20 min of moderate to vigorous physical activity daily, with a goal of 60 min, all in the context of a calorie-controlled diet (20). The most recent American Guidelines on physical activity confirm the AAP and the Endocrine Society recommendations for children and adolescents (Table 2) (39). In severe obese subjects exercises that cause constant weight or repeated impact on the child's legs, feet, and hips should be avoided (22).

**Table 2:** Guidelines for preschool-aged children and for school-aged children and adolescents.

Preschool-aged children (3 through 5 years)	Preschool-aged children should be physically active throughout the day to enhance growth and development. Adult caregivers should encourage active play that includes a variety of activity types
School-aged children and adolescents (6 through 17 years)	Provide opportunities and encouragement to participate in physical activities appropriate for age and enjoyable. Children and adolescents should do 60 min (1 h) or more of moderate-to-vigorous physical activity daily
	**Aerobic**: Most of the 60 min or more per day should be either moderate- or vigorous-intensity aerobic physical activity and should include vigorous-intensity physical activity on at least 3 days a week
	**Muscle-strengthening**: As part of their 60 min or more of daily physical activity, children, and adolescents should include muscle-strengthening physical activity on at least 3 days a week
	**Bone-strengthening**: As part of their 60 min or more of daily physical activity, children, and adolescents should include bone-strengthening physical activity on at least 3 days a week

*Modified by (39)*.

## Other Lifestyle Modifications and Behavioral Treatment

Other than adopting healthy eating habits and increasing physical activity, the primary goal in prevention and treatment of obesity and MetS in pediatric population includes lifestyle modifications, such as limiting video exposition time (23, 40) and adopting healthy sleep habits. Disordered sleep length and quality affect appetite and decrease insulin sensitivity (41). The AAP recommends to limit screen time to <2 h/day (8). The National Sleep Foundation recommends 8–11 h of sleep for school age children and adolescents (42). Even independently by the severity of obesity, these additional behavioral modifications reduce the likelihood of developing MetS (20, 24).

Pediatrician should assess patients and families for readiness to change. This information can guide the type and degree of interventions and may help to avoid excess time investing in non-effective interventions (13). Programs involving the whole family in lifestyle modifications have more positive and durable effects compared to those directed at child alone (8, 35).

## Pharmacological and Surgical Treatment of Obesity

Pharmacotherapy of obesity in children is limited. There are poor evidences regarding the safety and efficacy of pharmacological agents aimed to weight reduction in adolescents, especially in the long term (43). When indicated, pharmacological therapies should be prescribed by experienced clinicians, who can offer close monitoring for weight reduction result and potential side effects. In addition, pharmacological option should be considered only after the proved failure of a formal program of lifestyle modification, which should however be continued in parallel with pharmacotherapy (20). Currently, the US Food and Drug Administration (FDA) indicates only Orlistat for weight loss in adolescents over 12 years of age (44). By inhibiting intestinal lipase, it reduces the absorption of triglycerides and cholesterol (45). It has frequent gastrointestinal adverse effects and can affect the absorption of fat-soluble vitamins (46). Pharmacological treatment efficacy is limited. At 6 and 12 months of follow-up, it determines, respectively, an average weight loss of 3 and 5% (47, 48). Weight loss agents, other than Orlistat, are still under investigation for the use in adolescents (16, 49, 50).

Bariatric surgery can result in significant short-term weight loss in obese adolescents, but it is reserved for the most severely affected subjects, who have already completed or almost completed their growth and have completed pubertal development (22, 51, 52). Surgical option is recommended only if BMI exceeds 40 kg/m^2^ with mild comorbidities or 35 kg/m^2^ with severe comorbidities. Severe comorbidities include type 2 diabetes mellitus (T2DM), moderate to severe sleep apnea, severe hepatic fibrosis resulting from non-alcoholic steatohepatitis (NASH), pseudotumor cerebri, or debilitating orthopedic problems. Mild comorbidities include dyslipidemia, hypertension, mild sleep apnea, moderate orthopedic problems, NASH, and psychological distress related to obesity (20, 22). Bariatric surgery should be considered only after the failure of a formal lifestyle modification program with documented compliance (20, 22). Prior to proceed with surgery, family competency should be evaluated, as well as should be excluded potential underlying psychiatric illnesses, substance abuse or eating disorders. The benefits of surgery option must be evaluated in the context of potential surgical complications (53). The research on the safety, efficacy, and long-term outcomes of bariatric surgery in adolescents is limited, but data on adults must be considered as surrogate evidence (54). Serious and short-term complications (i.e., abscess, infections, pulmonary embolism, etc.) can occur in 4.1% of all patients (53). A recent longitudinal study on a cohort of 161 adolescents reports 1.9% of mortality in the 5 years after surgery (55). Mild- and long-term complications, which include intestinal obstruction, ulcers and hernia, and metabolic complications (i.e., nephrolithiasis, hypoglycemia and vitamin, or mineral deficiencies) may also be considered but are hardly to monitor because of the high rate of dropout to the follow-up (53). Bariatric surgery in adolescents can result in improvement or resolution of comorbid conditions, such as T2DM, sleep apnea and hypertension, and can lead to good effect on weight loss (55, 56). However, further research is required because of the impact of the development of complications and the relatively poor number of trials which determine the long-term efficacy and safety in adolescents (53, 54, 57). Literature consistently reports that both surgeon and surgical center experience are predictors of safety and a team of specialists capable of long-term medical and psychological follow-up care for the patient is essential (20, 54).

## Treatment of MetS Risk Factors or Components Other Than Obesity

Treatment of childhood MetS include disease-specific management of its various components. Cardiovascular morbidity represents the effect of a continuum in the spectrum of the single CVR factors and a youth with multiple borderline risk factors might have equivalent risk of a person with extreme abnormality of a single major risk factor (8). The presence of any combination of multiple risk factors should prompt intensification of therapy (8).

## Hypertension

The overall aims of hypertension (HTN) treatment in pediatric population should include obtaining a blood pressure (BP) level that reduce the risk of target organ damage and reducing the risk of maintaining hypertension and related increasing CVR in adulthood (58). The new Guidelines for High Blood Pressure in children and adolescents, published in 2017, indicate as the goal of non-pharmacological and pharmacological therapy a BP <90th percentile or <130/80 mmHg in adolescents ≥13 years old (58).

Lifestyle intervention are the first-line approach recommended for HTN (58). There is good evidence in adults as in childhood that nutritional interventions, including reduced dietary sodium and increased olive oil polyphenols intake, result in lowering BP levels and cardiovascular mortality (59–61). In addition, higher intake of fruits and vegetables are also associated with lower BP and with lower risk of high BP in young adulthood (62, 63). Several studies conducted in children and adolescents support the evidence of the benefit of physical activity on BP (64), also independently from the type of exercise (aerobic training, resistance training or combined training) (34). AAP recommends sessions of 30–60 min of moderate to vigorous physical activity at least 3–5 days per week (58).

When HTN persists despite lifestyle modifications or in case of symptomatic HTN or stage II hypertension without modifiable factors, pharmacologic treatment should be initiated (58). Therapy should be started with a single medication at the low end of dosing range and can be titrated every 2–4 weeks. The patient should be seen every 4–6 weeks until BP has normalized (e.g., <90th percentile), than the frequency of visits can be extended to every 3–4 months (58). Treatment should be initiated with an ACE inhibitor, angiotensin receptor blocker (ARB), long acting calcium channel blocker or a thiazide diuretic. Studies completed in children do not find significant differences in the effectiveness between these different agents and show few adverse effects (65, 66). When hypertension is in combination with T2DM, ACE inhibitor or ARB are recommended as first-line therapy (8, 58, 67). Combination therapy may be required if HTN does not normalize on single agent therapy (58). In children requiring pharmacologic treatment, lifestyle changes should be continued, also in order to improve antihypertensive medications efficacy (58).

## Dyslipidemia

The association of obesity and hyperlipidemia is predictive of fatal and non-fatal cardiovascular events in adult life (68). The role of dietary fat intake in increasing cardiovascular risk is still debated (69, 70). A review of De Souza et al. reports no association between saturated fat intake and cardiovascular disease (69). In addition available evidence from randomized controlled trials shows that saturated fatty acids replacement with polyunsaturated fatty acids reduces serum cholesterol levels but not results in a lower risk of death from coronary heart disease (70). However, recent evidences support the importance of the type of fats consumed rather than total fat intake in influencing cardiovascular health and recommend to avoid industrially produced trans-fats (71).

The type of dyslipidemia associated with Mets is usually treated with lifestyle interventions only, but medications should be initiated when appropriate. The authors of the Expert Panel Guidelines for Cardiovascular Health and Risk Reduction published in 2011 provide the evidence based guidance for dietary management of dyslipidemia in children and adolescents (CHILD 1 and CHILD 2 approach) (8). A reduction of total fat between 25 and 30% of daily calories and of saturated fat <10% calories and a cholesterol intake <300 mg/day has been shown to safely and effectively reduce total cholesterol and LDL cholesterol (8). When indicated, more stringent diet restrictions have been shown to be safe and modestly effective (8).

In children with obesity and elevated triglyceride level, reduction of simple carbohydrates intake is also effective. The use of plant sterols or stanol esters is usually reserved for children with primary elevation of LDL cholesterol (8).

For children who will unlikely achieve lipid targets with behavioral modifications alone, pharmacologic treatment should be evaluated and it should always be proposed considering the context of complete CVR profile of the patient (8). To define the cut-off points at which the pharmacologic therapy should be considered the American Academy of Pediatrics refers to 1992 National Cholesterol Education Program Guidelines (72). Elevated triglycerides and low HDL cholesterol with relatively normal LDL cholesterol represent the specific features of dyslipidemia in MetS (5, 13). Pharmacological treatment is rarely necessary for children with elevated triglyceride levels that have a good response to weight loss and lifestyle changes. Increased fish intake or fish-oil supplementation may be considered (8). Initiation of medication therapy should be proposed on the basis of a specific step-intervention well described in the AAP Expert Panel Guidelines (8). In particular, the use of statin is indicated when a moderate hypertriglyceridemia (200–499 mg/dl−2.26–5.64 mmol/l) is associated with non-HDL cholesterol level above 145 mg/dl−3.76 mmol/l (8, 73). At present, FDA does not approve in children the use of other triglycerides level-lowering medications, such as fibrate and nicotinic acid (73).

## Glucose Impairments and Type 2 Diabetes Mellitus

Treatment of insulin resistance (IR) involves lifestyle modification only. The use of metformin to treat children and adolescents with IR but not abnormal glucose concentrations is uncommon. Although some beneficial effects of metformin on BMI reduction have been demonstrated, there is no clear evidence for long-term benefits (74–76). In obese adolescents impaired fasting glucose or impaired glucose tolerance are often transient and about 60% of them revert to normal glucose tolerance within 2 years as the IR of puberty is reduced (77). Less than 2% of European adolescents with glucose abnormalities develop type 2 diabetes mellitus (T2DM) in the next 5 years (78, 79). Therefore, there is currently no evidence base for the use of metformin or other medications for the pharmacological treatment of IR (67, 80).

Lifestyle intervention, including dietary and exercise changes, is also the core of treatment of T2DM. Healthy behavioral changes are essential for success also when pharmacological treatment is indicated and should be periodically checked. Initial treatment of youth with T2DM should include metformin and/or insulin alone or in combination, considering the symptoms, the severity of hyperglycemia and the presence or absence of ketosis/ketoacidosis (67). The goal of pharmacologic treatment is to obtain an HbA1c <7.0% (67).

When HbA1c is <8.5% (which corresponds to an estimated average glucose of 200 mg/dl) and the patient is asymptomatic and metabolically stable, treatment should be started with metformin with a target of HbA1c <7% within 4 months (67, 74). Metformin acts reducing hepatic gluconeogenesis and stimulating peripheral glucose uptake (80). Gastrointestinal symptoms (transient abdominal pain, diarrhea or nausea) are the most common side effects reported for metformin use (81). They generally resolve or ameliorate with time or with dose titration and they may be attenuated using extended release formulations (67). The risk of lactic acidosis is extremely low. Addition and titration of basal insulin and, if necessary, of prandial insulin, is recommended when patients fails to reach HbA1c target within 4 months of metformin monotherapy or if contraindications or intolerable side effects of metformin develop (67, 74).

When ketosis and/or ketoacidosis are present, intravenous or subcutaneous insulin treatment is recommended (67, 74). Once corrected the acute metabolic abnormality, a therapy with intermediate-acting or basal insulin once daily is usually effective. Metformin can also be started along with insulin therapy, also in order to achieve, within 4–6 weeks, effective metformin monotherapy (67).

Blood glucose self-monitoring should be performed regularly during therapy, individualizing the frequency and considering the available resources. Continuous glucose monitoring benefits are still not clearly examined in youth onset T2DM (67).

## NAFLD

Non-alcoholic fatty liver disease (NAFLD) represents the hepatic manifestation of MetS. The goal of treatment of pediatric NAFD is to stop liver injury and reverse the progress form steatosis to steatohepatitis, cirrhosis and its complications (82). The mainstay of treatment for NAFLD remains lifestyle modifications and weight loss. Probiotics and ω-3 fatty acids may ameliorate disease progression (82). Two different double-blind randomized trials demonstrate the favorable effects of probiotics (Lactobacillus GG and VLS#3) in obese children with steatosis (83, 84). However, long-term follow-up studies are necessary to confirm these results. In NAFLD experimental models the use of ω-3 fatty acids showed benefits in reducing liver steatosis and ameliorating insulin sensitivity and markers of inflammation (85), but clinical trials in humans are still contradictory (86). Although Vitamin E does not appear to be effective in improving liver histology and reducing aminotransferase levels (87), it is able to improve hepatocellular ballooning in about 40% of patients with NASH or borderline NASH (88), so it may be considered as a NASH-specific therapy in children (82).

## Conclusions

Although there is still a lack of consensus on how to define MetS and its components in pediatric population, early identification and treatment of obese children and adolescents with multiple metabolic derangements allows to concentrate resources, particularly for children at higher risk, and to target focused intervention aimed to reduce the risk of cardiometabolic disease. Although sometimes the diagnosis is delayed because MetS, as obesity, is underperceived by families, general pediatricians and other pediatric sub specialists, early identification, and management are crucial and may help to attenuate the disease process. An expert multidisciplinary team, which includes pediatrician, mental health practitioner, dietician, nurses, and other referral specialists for the single complications is pivotal, both at the level of prevention and therapy, also in order to offer individualized treatment (8, 22, 67, 89). The economic sustainability of the multidisciplinary team is a challenge for the health system. A policy that involves more investments on this topic would be crucial (90, 91).

## Author Contributions

EF and CM gave a substantial contribution to the conception of the work, the acquisition, analysis, or interpretation of data for the work, provide approval for publication of the content, and agree to be accountable for all aspects of the work in ensuring that questions related to the accuracy or integrity of any part of the work are appropriately investigated and resolved. EF wrote the manuscript and CM revised it critically for important intellectual content.

### Conflict of Interest

The authors declare that the research was conducted in the absence of any commercial or financial relationships that could be construed as a potential conflict of interest.
